# A Reversible Cytotoxic Lesion of the Corpus Callosum Developing after a Rapid Alteration in Cerebrospinal Fluid Pressure/Volume in a Patient with New Daily Persistent Headache

**DOI:** 10.1155/2020/8849645

**Published:** 2020-10-23

**Authors:** Todd D. Rozen, Hector A. Robles

**Affiliations:** Department of Neurology and Neuroradiology, Mayo Clinic Florida, Jacksonville, USA

## Abstract

A case is presented of a woman with a history of daily persistent head pressure and dizziness who developed a cytotoxic lesion of the splenium of the corpus callosum after an acute withdrawal of chronic acetazolamide treatment and then, in quick succession, a CSF pressure/volume drop with a lumbar puncture. This is the first documentation that rapid alterations of CSF pressure/volume may trigger cytotoxic lesions in the central nervous system.

## 1. Introduction

Cytotoxic lesions of the corpus callosum have been associated with multiple conditions. In many cases, these lesions are reversible. The exact etiology for these lesions is unknown but may involve cytokine activation. A case is presented of a woman with a history of daily persistent head pressure and dizziness who developed a cytotoxic lesion of the splenium of the corpus callosum after an acute withdrawal of chronic acetazolamide treatment and then, in quick succession, a cerebrospinal fluid (CSF) pressure/volume drop with a lumbar puncture. This is the first documentation that rapid alterations of CSF pressure/volume may trigger the development of cytotoxic lesions in the central nervous system.

## 2. Case Presentation

**Figure 1:**
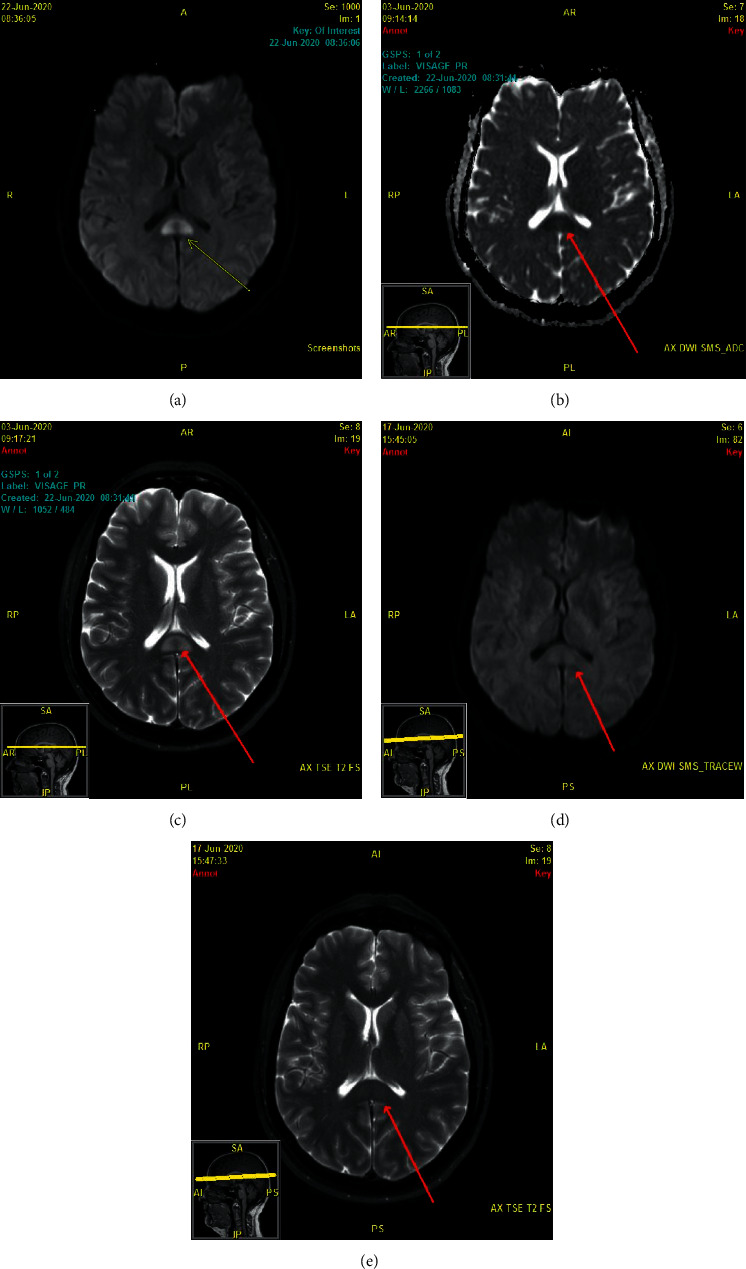
(a–c) MRI brain completed 16 days after lumbar puncture demonstrating an oval-shaped focus of abnormal unenhancing T2/FLAIR hyperintensity with restricted diffusion of the splenium of the corpus callosum (arrow). (a) Axial diffusion. (b) ADC mapping confirms true restricted diffusion. (c) Axial T2. (d, e) MRI brain completed 14 days after initial MRI with near-to-complete resolution of the corpus callosum lesion (arrow). (d) Axial diffusion. (e) Axial T2.

A 48-year-old woman presented with a two-year history of persistent head pressure and dizziness. She awoke one day with the symptoms. She had no prior headache history. She described her symptoms as holocranial pressure with some pain along with persistent lightheadedness. She would also experience true episodes of intermittent vertigo. She did not recognize a triggering event for her headaches. She did have some migraine-associated symptoms with exacerbations of head pressure including nausea, photophobia, and phonophobia. She had consulted with multiple physicians without a true diagnosis or effective treatment. There was some consideration for vestibular migraine, but migraine preventives (topiramate, valproic acid, and nortriptyline) were ineffective. Prior imaging demonstrated a partial empty sella, with no Chiari malformation or tonsillar ectopia. She had no vessel imaging studies. Past medical history was significant for noninsulin-dependent type II diabetes mellitus and hyperlipidemia. Her BMI was slightly elevated at 28. Neurologic examination was nonfocal including no disc edema on fundus evaluation. Her head pressure, however, worsened in the Trendelenburg position (10 degree head-down tilt). MR angiography and venography were completed and were normal. Formal neuro-ophthalmologic evaluation was normal with no evidence of disc edema. She was started on acetazolamide and did extremely well with almost complete alleviation of all of her symptoms at a dose of 1,125 mg per day of the short-acting formulation. She was given a diagnosis of new daily persistent headache (NDPH) based on the International Classification of Headache Disorders criteria as her headache/pressure symptoms began one day out of the blue and lasted for longer than 3 months [1]. The underlying etiology was hypothesized to be an abnormal reset of CSF pressure/volume to an elevated state because of her worsening in the Trendelenburg position (which rapidly increases intracranial CSF pressure within seconds of head-down tilt) and her response to CSF pressure/volume-lowering medications. This subtype of NDPH has been previously described by one of the authors (TDR) [2, 3]. After trying to switch her to the extended release formulation of acetazolamide, she lost control of her headaches/head pressure. A lumbar puncture (LP) was carried out on a semiemergent basis, and thus, she was still on a tapering dose of short-acting acetazolamide (750 mg) at the time of the procedure. The LP was completed to help establish if CSF pressure/volume was indeed the cause of her head pressure syndrome and to hopefully alleviate her symptom exacerbation. Opening pressure in the prone position was 23 cm of H_2_O, and closing pressure was 15 cm H_2_O after 15 mL of CSF was removed. CSF analysis was negative. The patient felt immediately better after the LP, but a day after, she developed an orthostatic headache which did not respond to conservative measures including tapering off the acetazolamide. An epidural blood patch alleviated the post-LP headache, but this seemed to reset her CSF pressure high again as she was back to daily head pressure/pain and dizziness. Her opening pressure fell within the new guidelines of normal, but she was on acetazolamide at the time of the LP, and she improved with CSF removal [4]. Eventually, she stabilized again on chronic acetazolamide therapy (1,125 mg per day) and did well for one year. She then had a second exacerbation of head pressure that was unresponsive to an increased dosing of her medication. Therapeutic LP was arranged. One day prior to her LP, she acutely stopped acetazolamide on her own volition. Opening pressure was 22 cm H_2_O, and closing pressure was 13 cm H_2_O after 15 cc of CSF was removed. The patient felt remarkably better after the procedure. Her only neurologic symptom was a two-hour episode of facial numbness and contralateral hemibody numbness which occurred two days after the LP. She did not develop a post-LP headache. Her CSF analysis demonstrated no evidence of inflammation with only one nucleated cell and a normal total protein and glucose level. Her Gram stain and bacterial and fungal cultures were also negative. A brain MRI was completed 16 days after the LP to look for any secondary causes for her worsening head pressure, and this demonstrated a cytotoxic edematous lesion in the splenium of the corpus callosum (Figures 1(a)–1(c)). Laboratory studies (CBC and basic metabolic panel) were normal at the time of neuroimaging. Serum cytokine levels were not obtained. No additional neurologic testing (such as an EEG) was deemed necessary as the patient was having no neurologic symptoms at the time of the MRI, and her neurologic examination was normal. She clinically was still much improved in regard to her head pressure. Repeat imaging 2 weeks later noted near-to-complete resolution of the lesion (Figures 1(d) and 1(e)). In retrospect, it would have been helpful to obtain serum cytokine levels at the time of the abnormal MRI imaging to see if they were elevated. However, measuring cytokine levels in the CSF would have been even more sensitive to look for alterations within the central nervous system.

The patient provided written consent for the use of her images.

## 3. Discussion

The cytotoxic, typically reversible, lesions of the corpus callosum have an unknown etiology. One of the current theories is that a release of cytokines including tumor necrosis factor-alpha (TNF‐*α*), interleukin‐1 (IL‐1), and interleukin‐6 (IL‐6) leads to a cascade of events which include microglial activation, increased glutamate synthesis with a rise in extracellular glutamine levels, mitochondrial dysfunction, and finally an influx of water into both glia and neurons causing cytotoxic edema [5, 6]. The splenium may be more susceptible to this process than the rest of the corpus callosum as it has a dual circulation and a higher abundance of cytokine and glutamate receptors [5]. Various triggers for the cytotoxic lesions including medications (typically antiepileptics: carbamazepine, lamotrigine, and phenytoin), infections (viruses: Epstein–Barr and adenovirus), CNS malignancies, subarachnoid hemorrhage, acute altitude sickness, metabolic disorders (acute renal failure, hypernatremia, hypoglycemia, and hepatic encephalopathy), and even several cases of migraine with aura have been reported [6, 7]. As our patient had none of these recognized factors, it can be theorized that a rapid alteration in CSF pressure/volume, first from acute withdrawal of chronic acetazolamide treatment with a presumed rise in CSF pressure/volume and then in quick succession a rapid CSF pressure/volume depletion with the LP, triggered cytokine release leading to various osmotic shifts resulting in the cytotoxic lesion.

Studies in the medical literature may help establish an association between rapid CSF pressure/volume alterations, cytokine activation, and cytotoxic lesions of the corpus callosum in patients with daily persistent headache from elevated CSF pressure. There are reports that patients with increased CSF pressure (idiopathic intracranial hypertension) have elevated levels of cytokines in their CSF including TNF-alpha and IL-6, but other studies have refuted this [8]. In addition, patients with NDPH have been shown to have elevated CSF TNF-alpha levels; thus, this could suggest that individuals with this unique headache syndrome are more susceptible to developing cytotoxic lesions of the corpus callosum from rapid alterations in CSF pressure/volume because they have cytokine activation at baseline [9]. Finally, after surgery to repair spinal fluid leaks, the levels of TNF-alpha, IL-6, and IL-10 dramatically increase in the CSF [10]. The cytokine release is suggested to be primarily secondary to the surgery itself, but a shift in CSF pressure/volume may also be involved in the cytokine activation.

## 4. Conclusion

From the case presentation, it is suggested that CSF pressure/volume-modulating medications should be tapered slowly if there is chronic usage and to avoid a rapid reduction in CSF volume with lumbar puncture in the setting of acute withdrawal of these medications, as this may cause a cytotoxic insult to the corpus callosum. Our patient had minimal neurologic manifestations, but these lesions have been associated with a mild-to-severe encephalopathy, paresis/paresthesias, and severe headaches [6].

## Data Availability

The data used to support the findings of this study are available within the article.

## References

[1] Headache Classification Committee of the International Headache Society (IHS) (2018). The international classification of headache disorders. *Cephalalgia*.

[2] Rozen T. D. (2018). A new subtype of chronic daily headache presenting in older women. *Journal of Women’s Health*.

[3] Rozen T. D. (2019). New daily persistent headache (NDPH) triggered by a single Valsalva event: a case series. *Cephalalgia*.

[4] Friedman D. I., Liu G. T., Digre K. B. (2013). Revised diagnostic criteria for the pseudotumor cerebri syndrome in adults and children. *Neurology*.

[5] Tetsuka S. (2019). Reversible lesion in the splenium of the corpus callosum. *Brain Behaviour*.

[6] Starkey J., Kobayashi N., Numaguchi Y., Moritani T. (2017). Cytotoxic lesions of the corpus callosum that show restricted diffusion: mechanisms, causes, and manifestations. *Radiographics*.

[7] Lin F. Y., Yang C. Y. (2011). Reversible splenial lesion of the corpus callosum in migraine with aura. *Neurologist*.

[8] Dhungana S., Sharrack B., Woodroofe N. (2009). IL-1*β*, TNF and IP-10 in the cerebrospinal fluid and serum are not altered in patients with idiopathic intracranial hypertension compared to controls. *Clinical Endocrinology (Oxf).*.

[9] Rozen T., Swidan S. Z. (2007). Elevation of CSF tumor necrosis factor alpha levels in new daily persistent headache and treatment refractory chronic migraine. *Headache*.

[10] Tang J. X., Baranov D., Hammond M., Shaw L. M., Eckenhoff M. F., Eckenhoff R. G. (2011). Human alzheimer and inflammation biomarkers after anesthesia and surgery. *Anesthesiology*.

